# Different degrees of expression of pulmonary fibrosis signs with intratracheal administration of bleomycin at different doses in male SD rats (a study aimed at helping to select the optimal bleomycin dose for modeling pulmonary fibrosis)

**DOI:** 10.3389/fphar.2025.1702991

**Published:** 2025-11-28

**Authors:** Elena A. Tukhovskaya, Yulia A. Palikova, Maria S. Severyukhina, Alina M. Ismailova, Victor Palikov, Gulsara A. Slashcheva, Natalya A. Borozdina, Evgeniy S. Mikhaylov, Irina N. Kravchenko, Vitaly Kazakov, Ekaterina N. Kazakova, Elena A. Kalabina, Ekaterina A. Rasskazova, Vladimir A. Rykov, Olga Patsap, Alexander A. Globenko, Ekaterina A. Jain, Andrey Kolosov, Arkady N. Murashev

**Affiliations:** 1 Shemyakin-Ovchinnicov Institute of Bioorganic Chemistry (Branch), Russian Academy of Sciences, Pushchino, Russia; 2 Peoples’ Friendship University of Russia named after Patrice Lumumba, Moscow, Russia; 3 Valenta Pharm JSC, Moscow, Russia

**Keywords:** pulmonary fibrosis, rats, bleomycin, general health status, dose-dependence, lung, respiratory function, histological assessment

## Abstract

**Background:**

Pulmonary fibrosis (PF) is a life-threatening lung disease. PF develops under the influence of various damaging agents. To study new therapeutic strategies, a PF model with intratracheal administration of bleomycin (BLM) is used.

**Methods:**

We studied the effect of different doses of BLM. The study was performed on male SD rats. BLM was administrated at doses of 0.5, 1.5, 2.5, 3, and 5 mg/kg. Animals were observed for 21 days for the following parameters: the overall health status, body weight gain, and external respiratory function. On the 22nd day, the lung weight, cellular composition of the bronchoalveolar fluid (BALF), and hydroxyproline content were determined, and the degree of lung fibrosis was histologically assessed.

**Results:**

With an increase in the BLM dose, the overall health status deteriorates, the function of external respiration worsens, BALF neutrophilic infiltration increases, and PF severity increases. The least marked PF manifestations were observed after the administration of BLM at a dose of 0.5 mg/kg, and the most marked manifestations were observed after the administration of BLM at a dose of 5 mg/kg.

**Conclusion:**

Results obtained in the study demonstrate a dose-dependent effect of BLM on the PF severity, which provides information that will help select BLM dose suitable for obtaining desired degree of PF in animal models.

## 1 Introduction

Pulmonary fibrosis (PF) is a severe, life-threatening lung disease characterized by sudden onset and rapid, inexorable progression (Li et al., 2024; Richeldi et al., 2020) that often develops as a consequence of pneumonia, interstitial lung pneumonia, and acute respiratory distress syndrome (Travis et al., 2013; Ware and Matthay, 2000). Globally, PF affects approximately 5 million individuals (Kreuter et al., 2022). The 5-year survival rate is only 20%–25% after PF diagnosis (Lederer and Martinez). Overall, the etiology of PF development remains unclear, and PF is most often characterized by the term “idiopathic” (IPF) (Taskar and Coultas, 2006; Chen et al., 2023). Numerous epidemiological studies have shown the contribution of environmental factors to the risk of developing PF, such as exposure to metal dust (Gandhi et al., 2023). Thus, in Japan, over a 12-year period of studying postmortem changes in the lungs, PF phenomena were identified in workers at metallurgical plants, agricultural workers, poultry workers, miners, and people working with wood, organic solvents (Iwai et al., 1994; Baumgartner et al., 2000; Miyake et al., 2005), and metal dust (Harris et al., 2001). In postmortem studies conducted in Japan, patients with PF were found to have higher levels of inorganic particles such as silicon and aluminum in the pulmonary hilum lymph nodes (Kitamura et al., 2007). A multicenter study conducted in Korea in 2017 found that exposure to metal dust or any harmful environmental impact for more than 1 year was consistently associated with IPF (Koo et al., 2017). European studies have shown that air pollution, including excess levels of gases such as NO_3_, NO_2_, and O_3_—mainly from vehicle exhaust gases—increases the incidence and exacerbation of PF (Conti et al., 2018). Smoking significantly increases the incidence of PF (Kärkkäinen et al., 2017). Drugs are among the most common causes of PF. The greatest contribution to the development of PF, based on data from the top 10 countries that submitted spontaneous case reports (SRs) from 2019 to 2024 from the National Pharmacovigilance database (Canada, United States, Japan, Germany, Russia, France, Brazil, Argentina, United Kingdom, and China), is made by drugs from the antineoplastic and immunomodulating agent group—particularly rituximab (51.9% of all SRs)—followed by systemic hormonal preparations excluding sex hormones and insulins (7.4%) and agents for the respiratory system (7.1%) (Butranova et al., 2024). As PF is a serious lung disease that leads to decreased quality of life, disability, and rapid death, the search for new drugs for the treatment of PF is a major task in pharmaceutical and medical sciences. Animal models of PF are used to study the effectiveness of new therapeutic agents for the treatment of PF, particularly modeling using intratracheal administration of the antitumor glycopeptide antibiotic, bleomycin (BLM). The mechanism of BLM action is based on DNA fragmentation and the destruction of its spiral structure, which leads to the inhibition of cell fission (Cheson et al., 2001). One of the side effects of BLM is pulmonary toxicity, particularly the development of PF (De Lena et al., 1972; Blum et al., 1973; Azambuja et al., 2005). It was previously established that the incidence of pulmonary complications in people receiving BLM antitumor therapy depends on the dose of BLM received, the route of administration, the scheme and frequency of administration (Haas et al., 1976; Krakoff et al., 1977; Cooper and Hong, 1981), and the accumulation of BLM (Willson, 1978; Collis, 1980; Hay et al., 1991). In preclinical practice, pulmonary toxicity of BLM is used to model PF in rats and mice with intravenous and intratracheal administration. However, with intravenous administration, PF develops over a long period of time and is not as reproducible as with intratracheal administration (Della Latta et al., 2015). The BLM-induced PF model is the gold standard in preclinical studies of therapeutic agents’ efficacy. For example, the efficacy of drugs approved by the FDA as effective in PF treatment, namely, nintedanib and pirfenidone, which were approved by the FDA on 15 October 2014 (a; b), was initially studied using this model (Wollin et al., 2014; Inomata et al., 2014; Kehrer and Margolin, 1997; Schelegle et al., 1997). To model PF in mice and rats, different investigators use intratracheal administration of BLM in different doses: 0.3 (Principi et al., 2023), 0.8 (Hung et al., 2022), 1 (Prêle et al., 2022), 1.5 (Chen et al., 2024), 2 (Bonatti et al., 2023), 2.5 (Kadam and Schnitzer, 2024a), 3 (Xie et al., 2023), 5 (Liang et al., 2024), 7.5 (Santos-Ribeiro et al., 2023), 8 (Chu et al., 2019), and 10 mg/kg (Ge et al., 2024). Studies using multiple doses of BLM are very limited. There are very few studies where research workers use multiple doses of BLM; for example, Kim et al. (2010) performed studies in mice, which showed that different doses of BLM can have different effects. In addition, Kadam et al. (Kadam and Schnitzer, 2024a; Kadam and Schnitzer, 2024b) administered several doses of BLM to mice and rats, and an extremely high sensitivity of mice to a dose of BLM of 3 mg/kg was observed. The animals were studied for the expression of various inflammation inhibitors, signaling peptides, and proteins, and the signaling pathways that contribute to the development of the fibrotic phenotype at different stages of PF development were studied. Because there are so many rodent PF modeling studies using only a single dose of BLM, with the doses varying across studies, it is difficult for research workers to select the optimal dose of BLM, so we chose to conduct a study using multiple doses of BLM simultaneously to determine whether the severity of PF is dependent on the dose of BLM noxious agent administered directly into the trachea. Our ultimate goal is to enable research workers modeling PF to select the desired degree of lung injury that suits their specific research goals using a widely available and inexpensive arsenal of methods.

## 2 Materials and methods

### 2.1 Animals

The study used 60 mature male SD rats that were 8–10 weeks old. Animals were obtained from the Pushchino Nursery of Laboratory animals (Pushchino, Russia). All animal procedures and manipulations were approved by the Committee for Control over Care and Use of Laboratory Animals of BIBCh RAS (IACUC) (protocol number 936/23 from 24.04.2023) and were carried out in accordance with the EU Directive 2010/63/EU. After receiving from the nursery, the animals were adapted within 7 days. During the adaptation period, the health status of the animals was monitored through clinical examination. Animals with no signs of health problems were selected for the experiment. Animals were randomly divided into groups using body weight as a criterion so that the average weight of animals did not differ between the groups. Each animal was assigned an individual number, according to which the animal was marked with a puncture of the auricle. During the study, the animals were kept under controlled environmental conditions in a barrier zone with a “clean” and “dirty” corridor system with controlled environmental conditions (temperature 20 °C–24 °C, relative humidity 30%–55%, 12 h light cycle; 08:00–20:00—“day,” 20:00–08:00—“night”; 10-fold change in air volume in the room per hour). The animals received *ad libitum* Velaz FORTI 1324 Maintenance Diet as food (Altromin Spezialfutter GmbH & Co KG, Im Seelenkamp 20, D-32791 Lage, Germany).

### 2.2 Design description

The animals were divided into groups of 10. The animals were modeled for PF by intratracheal administration of BLM solution in different doses. Animals of the first group were administered saline (VEH). Animals of the second, third, fourth, fifth, and sixth groups were administered BLM at doses of 0.5, 1.5, 2.5, 3, and 5 mg/kg, respectively. The injection volume was 0.5 mL/kg (Table 1). Before administration (day 0) and on days 3, 7, 14, and 21 after PF modeling, the following parameters were determined in animals: clinical signs of health abnormalities, body weight, feed consumption, and respiratory parameters. In the presence of significant clinical abnormalities, the clinical signs of health abnormalities were recorded daily. If animals developed abnormalities incompatible with life, the animals were subjected to unplanned euthanasia and necropsy with lung sampling. At the end of the in-life phase, on the 22nd day after BLM administration, the animals were euthanized under anesthesia with a mixture of Telazol® (tiletamine + zolazepam) + xylazine, followed by exsanguination, and the lungs were collected, cleaned, and weighed. The left lung was subjected to bronchoalveolar lavage (BAL), and the right lung was fixed for subsequent histological analysis. After BAL, the left lung of six animals from each group was frozen at −70 °C for subsequent hydroxyproline ELISA. For the remaining four animals, the left lung was fixed for further histological evaluation along with the right lung.

**TABLE 1:** Groups and doses.

Group number	Drug	BLM dose, mg/kg	Route of administration, volume of administration, ml/kg	Animal numbers
1	VEH	-	Intratracheal bolus administration, 0.5	1–10
2	BLM	0.5		11–20
3		1.5		21–30
4		2.5		31–40
5		3		41–50
6		5		51–60

### 2.3 PF modeling

**FIGURE 1:**
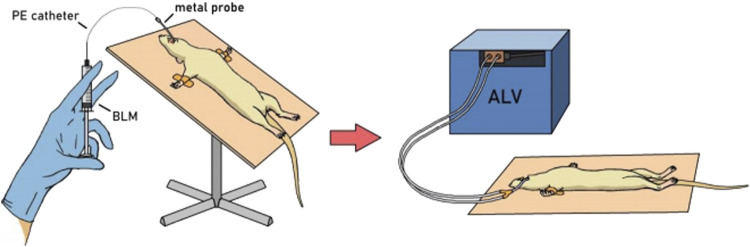
PF modeling in a male SD rat.

PF was modeled as described previously (Tukhovskaya et al., 2024) by a single intratracheal administration of BLM solution in different doses (Table 1) in a volume of 0.5 mL/kg followed by hyperventilation. The procedure was performed on animals anesthetized with an intramuscular injection of a mixture of Telazol® + Xyla® at a dose of 40 mg/kg + 10 mg/kg. The animals were fixed on a raised operating table in a supine position by the hind legs and upper incisors. The oral cavity was opened, and a metal probe with a ball on the end was inserted along the surface of the tongue. On exhalation, overcoming the resistance of the vocal cords, the probe was inserted into the trachea. A polyethylene catheter (PE-10, outer diameter 0.60 mm) was inserted into the probe opening and introduced to a premeasured depth (from the incisors to the middle of the sternum). A syringe with the BLM solution (or saline in the control group) was attached to the catheter and, holding the animal in a vertical position, the BLM solution was slowly injected. Immediately after intratracheal administration of BLM or saline, hyperventilation of the lungs was performed. A Ugo Basile 7025 artificial lung ventilation apparatus was connected to the probe located in the trachea (Figure 1). The following hyperventilation parameters were set: tidal volume 30 mL/kg, respiratory rate 60 times/min, and exposure time 10 min. After completion of the procedure, the animals were returned to the holding cage under constant observation until recovery from anesthesia.

### 2.4 Drugs

To model PF, we used the pharmaceutical drug (bleomycin hydrochloride) manufactured by JSC Omutninskaya Scientific Experimental Industrial Base, Russia. The drug was a lyophilizate in a glass vial. The BLM solution for administration was prepared immediately before use by adding a calculated amount of water for injection to the vial to obtain a stock solution with a concentration of 10 mg/mL. This stock solution was used to administer BLM at a dose of 5 mg/kg. To prepare BLM solutions for doses of 3, 2.5, 1.5, and 0.5 mg/kg, the stock solution was diluted with water for injection by 1.7, 2, 3.3, and 10 times, respectively. The prepared BLM doses were administered to the animals immediately after preparation; any remaining solution was discarded.

To anesthetize animals during PF modeling and before necropsy, we used Telazol, Zoetis Manufacturing & Research Spain, S.L., Spain, mixed with Xyla, Interchemie werken “De Adelaar” BV, Estonia. The Telazol solution was prepared according to the instructions by adding 5 mL of the solvent (water for injection) to the vial containing the lyophilizate. The resulting solution was stored in the refrigerator (at 2 °C–8 °C) for no longer than 30 days. Xyla is available as a ready-to-use solution. After opening for the first time, the solution was stored in the refrigerator (at 2 °C–8 °C) for no longer than 30 days.

### 2.5 Registration of clinical signs of deviations in the health status of animals

The health status of the animals was assessed before group formation and on days 3, 7, 14, and 21 after PF modeling. In the presence of significant deviations in health, the health status of the animals was recorded daily. If deviations incompatible with life appeared in the animals, they were euthanized, and an autopsy was performed with the collection of lung samples.

### 2.6 Body weight and weight gain

Animals were weighed when forming groups and then on the third, seventh, 14th, and 21st days after modeling PF, but in the presence of pronounced health deviations, animals were weighed daily. Body weight gain was calculated relative to the body weight on day 1 of the study.

### 2.7 Food consumption

Food consumption was assessed by weighing the feed rack at the start of the 24-h food consumption recording before PF modeling and then on days 2–3, 6–7, 13–14, and 20–21.

### 2.8 External respiration parameters (spirometry)

The external respiration parameters of the animals (respiratory rate, tidal volume, and the maximum expiratory volume) were recorded before BLM administration on day 0 and then on days 2, 7, 14, and 21 after BLM administration using an FE141 Spirometer with PowerLab 8/35 computer system (ADInstruments Pty Ltd., Australia).

### 2.9 Euthanasia and necropsy

On the 22nd day after modeling, the animals were subjected to planned euthanasia under anesthesia with a mixture of Telazol® (tiletamine + zolazepam) + Xyla at doses of 40 mg/kg + 10 mg/kg, followed by total blood sampling from the inferior vena cava. During necropsy, the animal lungs and trachea were removed, clearing them of connective tissue.

### 2.10 Visual assessment of lung damage

The removed lungs were visually assessed for external signs of damage.

### 2.11 Lung weight

During necropsy, the lungs with trachea were weighed and the relative weight of the lungs relative to the body weight was calculated.

### 2.12 BAL procedure

The right bronchus was ligated, and BAL was performed to obtain the bronchoalveolar fluid (BALF). BAL was made by the infusion of 2 mL of PBS solution into the lung through the trachea and draining the resulting wash by gravity into a 15-mL test tube. The procedure was performed three times, and the total volume of PBS for BAL was 6 mL. The BALF was divided into two parts: one was used to count the total number of nucleated cells in a Goryaev’s chamber and the second was centrifuged to prepare a smear.

### 2.13 Counting of BALF cells in Goryaev’s chamber

The concentration of nucleated cells was calculated. For this purpose, a tenfold dilution of BALF in 4% acetic acid was performed to lyse the erythrocytes, the resulting sample was used to fill the Goryaev’s chamber, and the nucleated cells were counted per 100 cells.

### 2.14 Preparation of a BALF smear and cell counting

The BALF was centrifuged in an Eppendorf 5804 R centrifuge at 3,000 rpm at 4 °C for 15 min. The supernatant was collected, and a smear was prepared from the sediment after pipetting with 3 μL of fetal calf serum. The smear was stained according to Pappenheim. The cellular composition of the stained smears was analyzed: the percentage of alveolar macrophages, segmented neutrophils, band neutrophils, eosinophils, and lymphocytes was calculated per 100 cells.

### 2.15 Lung tissue sampling and preparation for hydroxyproline ELISA analysis

During necropsy, lungs from six animals in each group were taken from the animals along with the trachea. After performing BAL for the left lung, the left bronchus was ligated and the left lung was cut off. The left lung was divided into two parts, which were placed in weighed 2-mL Eppendorf tubes. Lung samples were frozen at −70 °C until the homogenate was prepared. To prepare the homogenate, the lung sample was grinded with a manual homogenizer MT 30K directly in the Eppendorf tube after defrosting. The tube with the homogenate was weighed, and the mass of the resulting homogenate was calculated by subtracting the mass of the empty tube from the mass of the tube with the homogenate. The homogenate was frozen at 70 °C, defrosted again, and PBS was added at 1:12 ratio of weight: volume. For the homogenate + PBS mixture, two cycles of freezing at −70 °C and thawing were performed to more effectively grind the tissue. After the last thawing, the sample was centrifuged at 3,000 rpm at 4 °C for 15 min and then at 5,000 rpm at 15 °C for 15 min. The obtained supernatant was concentrated in special JetSpin^TM^ Centrifugal Filter tubes, 5 mL, 100,000 MWCO, PES, non-sterile, BIOFIL, China. The tubes for concentration were prewashed with PBS solution according to the instructions. The supernatant from the sample + PBS was poured into the washed tube and centrifuged at 3,500 rpm. The centrifugation time for all the samples was different (from 35 to 135 min). The samples were centrifuged to a double volume relative to the homogenate mass (e.g., homogenate weight 0.3 g; centrifugation-concentration to a volume of 0.6 mL).

### 2.16 Lung hydroxyproline enzyme-linked immunosorbent assay (ELISA)

The hydroxyproline content in the sample was determined by ELISA using the Rat Hyp (hydroxyproline) ELISA Kit, ELK Biotechnology, using an immunological plate spectrophotometer Multiskan™GO 1.01.12, Thermo Scientific, and measurement was carried out at 450 nm.

### 2.17 Preparation and analysis of the histological specimens

The right lung for all the animals and additionally the left lung for four animals from each group were pre-filled with 10% neutral formalin solution, fixed in the above solution, washed in running tap water, dehydrated in alcohols of increasing concentration, and embedded in paraffin. Paraffin sections of 4 μm–5 μm thickness were stained using the Masson method (Masson Trichrome dye, Bio-Optica, Italy). Histological preparations were examined using conventional light microscopy on an AxioScope.A1 microscope (Carl Zeiss, Germany). Micrographs of the histological preparations were obtained using a high-resolution Axiocam 305 color camera (Carl Zeiss, Germany) using ZEN 2.6 lite software (Carl Zeiss, Germany). The severity of PF was assessed in paraffin sections stained with a specific Masson trichrome dye using a modified semi-quantitative eight-point Ashcroft scale (Wollin et al., 2014). The final PF severity was assessed as the average score of fibrotic changes in the lungs in all the assessed fields of vision and in all sections of the organ (for all lobes). To increase the reliability of semi-quantitative assessment and prevent biased assessment, the histologist was blinded and histological slides were coded, making it impossible to know which group the animals belonged to.

### 2.18 Statistics

Microsoft Excel and Statistica for Windows, version 7, were used for statistical processing of the experimental data. The normality of distribution of all measurement data was tested using the Shapiro–Wilk W test. The mean values (MEAN) and standard deviations (SD) were calculated for all numerical data. Between-group differences for repeated measurement data (body weight, weight gain, and spirometry) were assessed using repeated measures ANOVA with power testing using Duncan’s multi-stage *post hoc* test. Between-group differences for outcome-based data (BALF cytology, lung tissue hydroxyproline concentration, lung weight, and histological semi-quantitative assessment data) were assessed using one-way ANOVA with power testing using Duncan’s multi-stage *post hoc* test.

The chi-square test was used to analyze the intergroup differences in the frequency of manifestation of a sign (frequency of manifestation of clinical signs in health status deviations).

### 2.19 Ethical considerations

All procedures and manipulations with animals were approved by the Institutional Animal Care and Use Committee of BIBCh RAS (IACUC) (protocol number 936/23 from 24.04.2023) and were carried out in accordance with the EU Directive 2010/63/EU.

## 3 Results

### 3.1 Assessing animals’ general state of health

No mortality was observed in the study; however, two animals, one receiving BLM at a dose of 3 mg/kg and the other receiving BLM at a dose of 5 mg/kg, developed severe cachexia (weight loss of approximately 50% of the initial weight) by the 14th day after the PF modeling and were, therefore, euthanized. Table 2 presents the daily clinical observations for animals with health abnormalities. It is evident that as the BLM dose increases, the frequency, number, severity, and duration of health problems also increase, indicating that the worsening of the animals’ condition is dose-dependent.

**TABLE 2:** Summary results of daily clinical examination.

Group number	Drug, dose	Description of clinical sign	Number of animals with clinical signs	Number of animals with signs of abnormal health in the group (number out of 10)
1	Saline	-	0	0 of 10
2	BLM 0.5 mg/kg	Short-term weight loss up to day 4 after PF modeling	1	1 of 10
3	BLM 1.5 mg/kg	Short-term decrease in body weight up to the 4–5 days after PF modeling	2	3 of 10
		Long-term weight loss up to 11 days after PF modeling	1	
		Tremor	2	
4	BLM 2.5 mg/kg	Short-term decrease in body weight up to 4–7 days after administration	4	8 of 10 ***@@&
		Long-term weight loss up to 21 days after PF modeling	4	
		Tremor	4	
		Slouch	2	
5	BLM 3 mg/kg	Short-term decrease in body weight up to 4–7 days after administration	4	9 of 10 ***@@@&&
		Long-term weight loss up to 14–21 days after PF modeling	5	
		Tremor	6	
		Slouch	4	
		Piloerection	1	
		Chromodacryorrhea	1	
6	BLM 5 mg/kg	Short-term decrease in body weight up to 4–7 days after PF modeling	4	10 of 10 ***@@@&&&
		Long-term weight loss up to 14–21 days after PF modeling	6	
		Tremor	6	
		Slouch	4	
		Piloerection	3	
		Chromodacryorrhea	1	
		Wobbling	1	

***p ≤ 0.001 relative to the control group; ^@@^ p ≤ 0.01 and p ≤ 0.001 relative to BLM 0.5 group; ^&^ p ≤ 0.05, ^&&^ p ≤ 0.01, and ^&&&^ p ≤ 0.001 relative to BLM 1.5 group according to the chi-square test.

### 3.2 Body weight and weight gain

**FIGURE 2:**
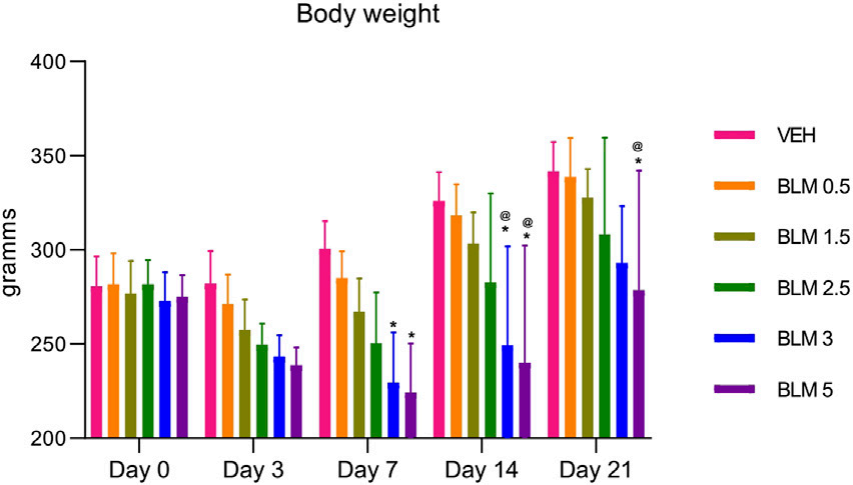
Dynamics of changes in the body weight of animals after administration of BLM in different doses.*p ≤ 0.05 relative to the control group and @ p ≤ 0.05 relative to the BLM 0.5 group according to the repeated measures test ANOVA with *post hoc* Duncan’s test. N for all groups = 10; for BLM 3 and BLM 5 groups on the 21st day of the study, N = 9. Data are presented as the MEAN ± SD.

The body weight of animals with BLM administration starting from the third day after PF modeling was significantly lower than that of the group receiving the vehicle saline. The dynamics of body weight reduction over the 21 days of observations indicates a dose-dependence of the BLM effect; that is, with an increase in the BLM dose, the body weight reduction was more pronounced (Figure 2).

**FIGURE 3:**
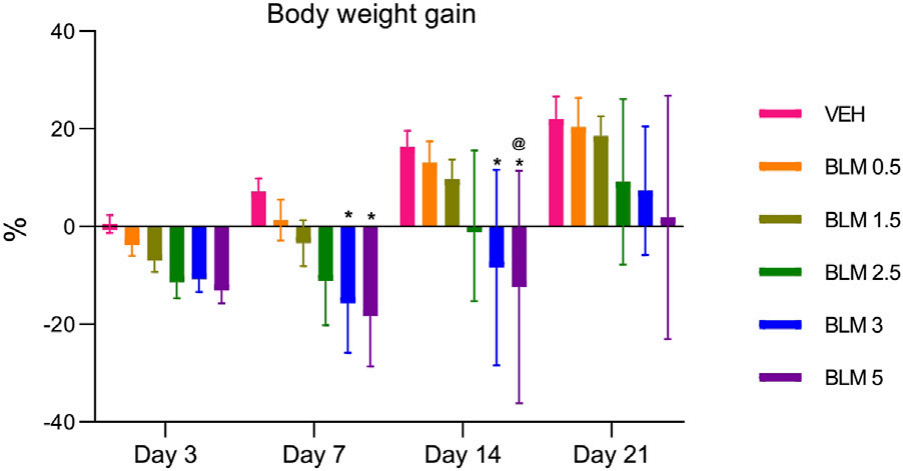
Dynamics of the body weight gain index of animals after PF modeling by the administration of BLM in different doses. *p ≤ 0.05 relative to the control group and ^@^ p ≤ 0.05 relative to the BLM 0.5 group according to repeated measures test ANOVA with *post hoc* Duncan’s test. N for all groups = 10; for BLM 3 and BLM 5 groups on the 21st day of the study, N = 9. Data are presented as the MEAN ± SD.

In all animals that were administered BLM, the body weight gain parameter decreased on the third day. The decrease in the body weight gain parameter was maintained on the seventh day in animals that received BLM at doses of 1.5, 2.5, 3, and 5 mg/kg and on the 14th day in animals that received BLM at doses of 2.5, 3, and 5 mg/kg. Starting from the 14th day, the animals showed a recovery increase in the body weight gain parameter; on the 21st day, the body weight gain parameter increased in all groups of animals. The dynamics of the body weight gain parameter indicates the dose-dependence of the BLM effect—with an increase in the BLM dose, the decrease in the body weight gain parameter is more pronounced and longer (Figure 3).

### 3.3 Food consumption

**FIGURE 4:**
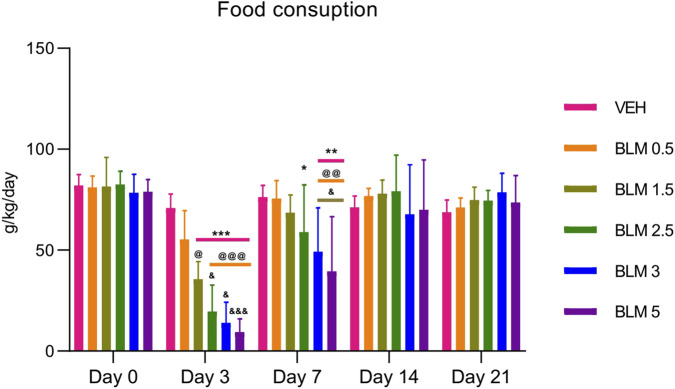
Dynamics of food consumption by animals after PF modeling by the administration of BLM in different doses. *p ≤ 0.05, **p ≤ 0.01, and ***p ≤ 0.001 relative to the control group; ^@^ p ≤ 0.05, ^@@^ p ≤ 0.01, and ^@@@^ p ≤ 0.001 relative to the BLM 0.5 group; ^&^ p ≤ 0.05 relative to the BLM 1.5 group according to repeated measures test ANOVA with *post hoc* Duncan’s test. N for all groups = 10; for BLM 3 and BLM 5 groups on the 21st day of the study, N = 9. Data are presented as the MEAN ± SD.

Food consumption is an indicator of the general health of animals. All animals showed a decrease in food consumption after PF modeling, and it was dose-dependent—an aggravation of the decrease in the indicator was observed with an increase in the BLM dose. A decrease in food consumption was observed until the seventh day after PF modeling, and from the 14th day, food consumption did not differ between the groups (Figure 4).

### 3.4 Evaluation of the external respiratory function—spirometry

**FIGURE 5:**
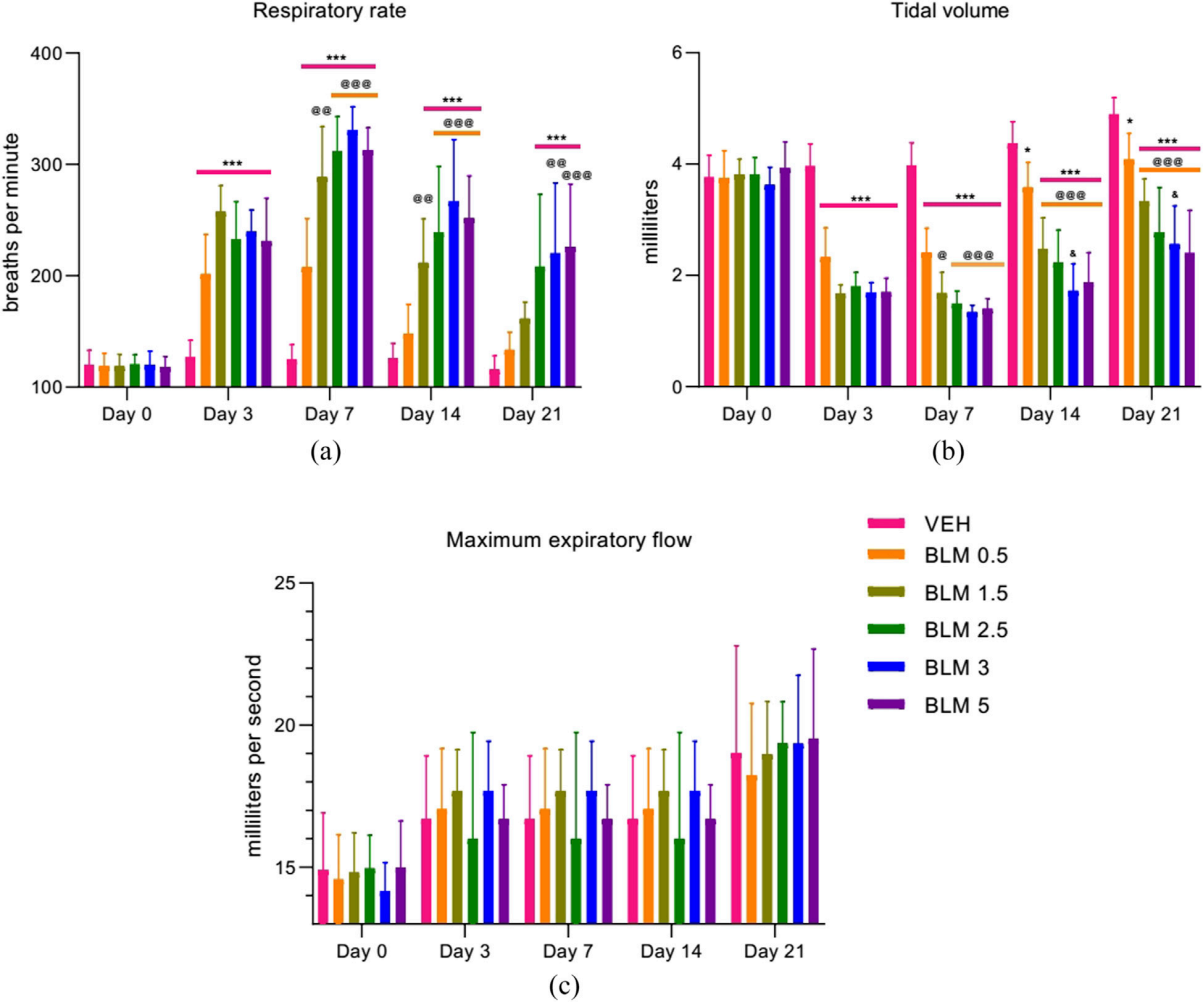
Dynamics of the external respiratory function parameters after PF modeling by the administration of BLM in different doses: **(a)** respiratory rate, **(b)** tidal volume, and **(c)** maximum expiratory flow. *p ≤ 0.05, **p ≤ 0.01, and ***p ≤ 0.001 relative to the control group; ^@^ p ≤ 0.05, ^@@^ p ≤ 0.01, and ^@@@^ p ≤ 0.001 relative to the BLM 0.5 group; ^&^ p ≤ 0.05 relative to the BLM 1.5 group according to repeated measures test ANOVA with *post hoc* Duncan’s test. N for all groups = 10; for BLM 3 and BLM 5 groups on the 21st day of the study, N = 9. Data are presented as the MEAN ± SD.

After PF modeling, the respiratory rate in all the animals increased significantly, and on the third day after modeling, the parameter did not differ between the groups with PF. Starting from the seventh day, the dose-dependence of the BLM effect was observed—with an increase in the BLM dose, the recovery of the respiratory rate over time was longer and less pronounced; however, recovery of the parameter was not complete in any of the groups (Figure 5a).

The tidal volume, a parameter related to the vital capacity of the lungs, significantly decreased in all animals with PF, with a subsequent tendency to recovery. At the same time, the dose-dependence of the effect of BLM on the parameter was observed starting from the third day after modeling—with increasing BLM dose, the recovery of the tidal volume over time was slower and less pronounced (Figure 5b).

The maximum expiratory flow rate significantly decreased on the third day after PF modeling. In the following days of the study, this parameter did not differ between the groups (Figure 5c).

### 3.5 Cellular composition of the BALF

After PF modeling, an increase in the total number of nucleated cells in the BALF was observed along with changes in the cellular composition of the BALF, and these changes varied depending on the BLM dose. The maximum contribution to the increase in the total number of cells in the groups receiving BLM at the lowest doses, namely, 0.5 and 1.5 mg/kg, was made by alveolar macrophages. Starting with a BLM dose of 2.5 mg/kg, the increase in the total number of cells in the BALF was due to both an increase in the number of alveolar macrophages and an increase in segmented neutrophils and lymphocytes (Table 3).

**TABLE 3:** Calculation of BALF cellular composition.

Parameter	Group 1 vehicle saline, n = 10	Group 2 BLM 0.5 mg/kg, n = 10	Group 3 BLM 1.5 mg/kg, n = 10	Group 4 BLM 2.5 mg/kg, n = 10	Group 5 BLM 3 mg/kg, n = 10	Group 6 BLM 5 mg/kg, n = 10
Counting of nucleated cells of the BALF in the Goryaev’s chamber						
Total cell concentration x10^5^/ml	2.35 ± 1.18	6.31 ± 2.12 *	5.00 ± 2.35 *	3.79 ± 1.81 #	4.46 ± 2.09	3.57 ± 1.55
BALF smear analysis						
Alveolar macrophages, %	98.28 ± 1.07	96.95 ± 2.35	95.00 ± 2.29 *	96.55 ± 2.64	93.10 ± 1.73 *#	83.71 ± 16.99 **#
Alveolar macrophages, ×10^5^/ml	2.32 ± 1.17	6.14 ± 2.09*	4.77 ± 2.31	3.66 ± 1.77 #	4.15 ± 1.92	2.95 ± 1.29
Band neutrophils, %	0.00	0.00	0.00	0.00	0.00	0.14 ± 0.27
Band neutrophils, ×10^5^ml	0.00	0.00	0.00	0.00	0.00	0.01 ± 0.02
Segmented neutrophils, %	0.15 ± 0.16	0.27 ± 0.52	0.95 ± 0.89 *	0.90 ± 1.23	2.38 ± 1.15 **@@	6.90 ± 11.05 **
Segmented neutrophils, ×10^5^/ml	0.00	0.01 ± 0.01	0.04 ± 0,05	0.04 ± 0.05	0.10 ± 0.06 **@@	0.21 ± 0.28 **
Eosinophils, %	0.00	0.00	0.05 ± 0.12	0.1 ± 0.1	0.10 ± 0.15	1.10 ± 1.94
Eosinophils, ×10^5^/ml	0.00	0.00	0.00	0.0	0.00	0.03 ± 0.05
Lymphocytes, %	1.57 ± 1.15	2.78 ± 1.90	4.00 ± 1.79 *	2.5 ± 1.5	4.42 ± 1.09 **	8.14 ± 9.80 **
Lymphocytes, ×10^5^/ml	0.03 ± 0,03	0.16 ± 0.11 **	0.19 ± 0.10 **	0.05 ± 0.12	0.21 ± 0.13 **	0.37 ± 0.63 **

* ≤0.05 and ** ≤0.01 relative to vehicle saline; @@ ≤ 0.01 relative to BLM 0.5 mg/kg group according to one-way ANOVA with *post hoc* Duncan’s test. Data are presented as the MEAN ± SD.

### 3.6 Hydroxyproline content in the lungs determined by ELISA

The hydroxyproline content was significantly increased in the groups receiving BLM at doses of 3 and 5 mg/kg compared with the control animals (Table 4).

**TABLE 4:** Hydroxyproline content in lung homogenates.

Group, n	Average concentration, ng/ml
Saline, n = 6	58.8 ± 0.5 ^$#^
BLM 0.5 mg/kg, n = 6	61.2 ± 1.5^$#^
BLM 1.5 mg/kg, n = 6	63.8 ± 1.7^$#^
BLM 2.5 mg/kg, n = 6	61.0 ± 2.3^$#^
BLM 3 mg/kg, n = 6	94.2 ± 18.9
BLM 5 mg/kg, n = 6	93.8 ± 11.0

^$^р ≤ 0.05 relative to the BLM 3 group, ^#^р ≤ 0.05 relative to the BLM 5 group according to one-way ANOVA with *post hoc* Duncan’s test. Data are presented as the MEAN ± SD.

### 3.7 Lung weight

**FIGURE 6:**
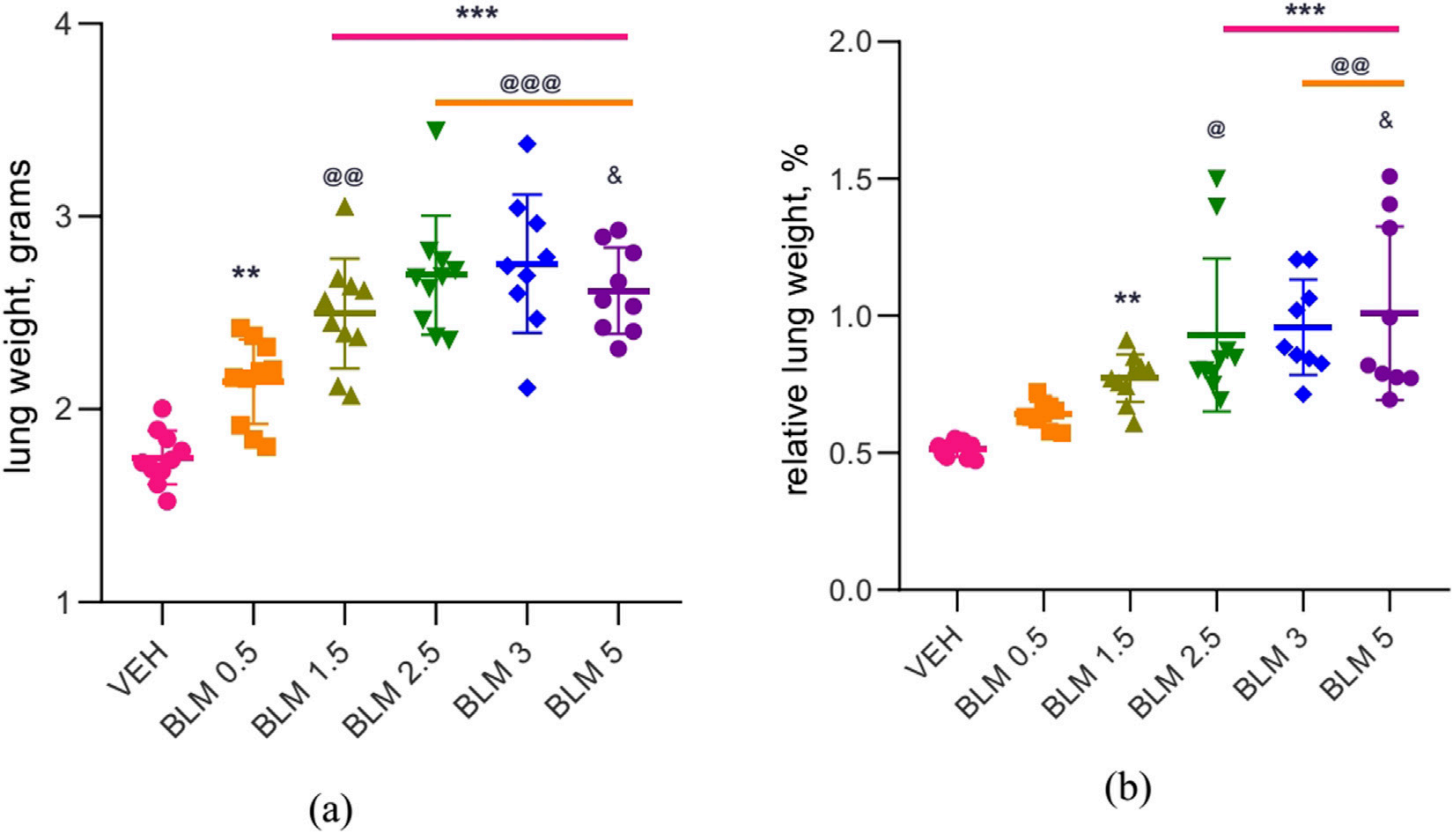
Lung weight in male SD rats on day 22 after PF modeling: **(a)** absolute lung weight, **(b)** relative lung weight relative to body weight at necropsy. ** р ≤ 0.01 and *** р ≤ 0.001 relative to saline; ^@^ р ≤ 0.05, ^@@^ р≤ 0.01, and ^@@@^ р≤ 0.001 relative to BLM 0.5 mg/kg group; ^&^ р≤ 0.05 relative to BLM 1.5 mg/kg group according to one-way ANOVA with *post hoc* Duncan’s test. For groups VEH, BLM 0.5, BLM 1.5, and BLM 2.5, N = 10; for groups BLM 3 and BLM 5, N = 9. Data are presented as the MEAN ± SD.

After PF modeling, all animals showed an increase in the absolute and relative lung weight, which was least pronounced in the group receiving BLM at a minimum dose of 0.5 mg/kg (Figure 6).

### 3.8 Visual assessment of the lung condition

**FIGURE 7:**
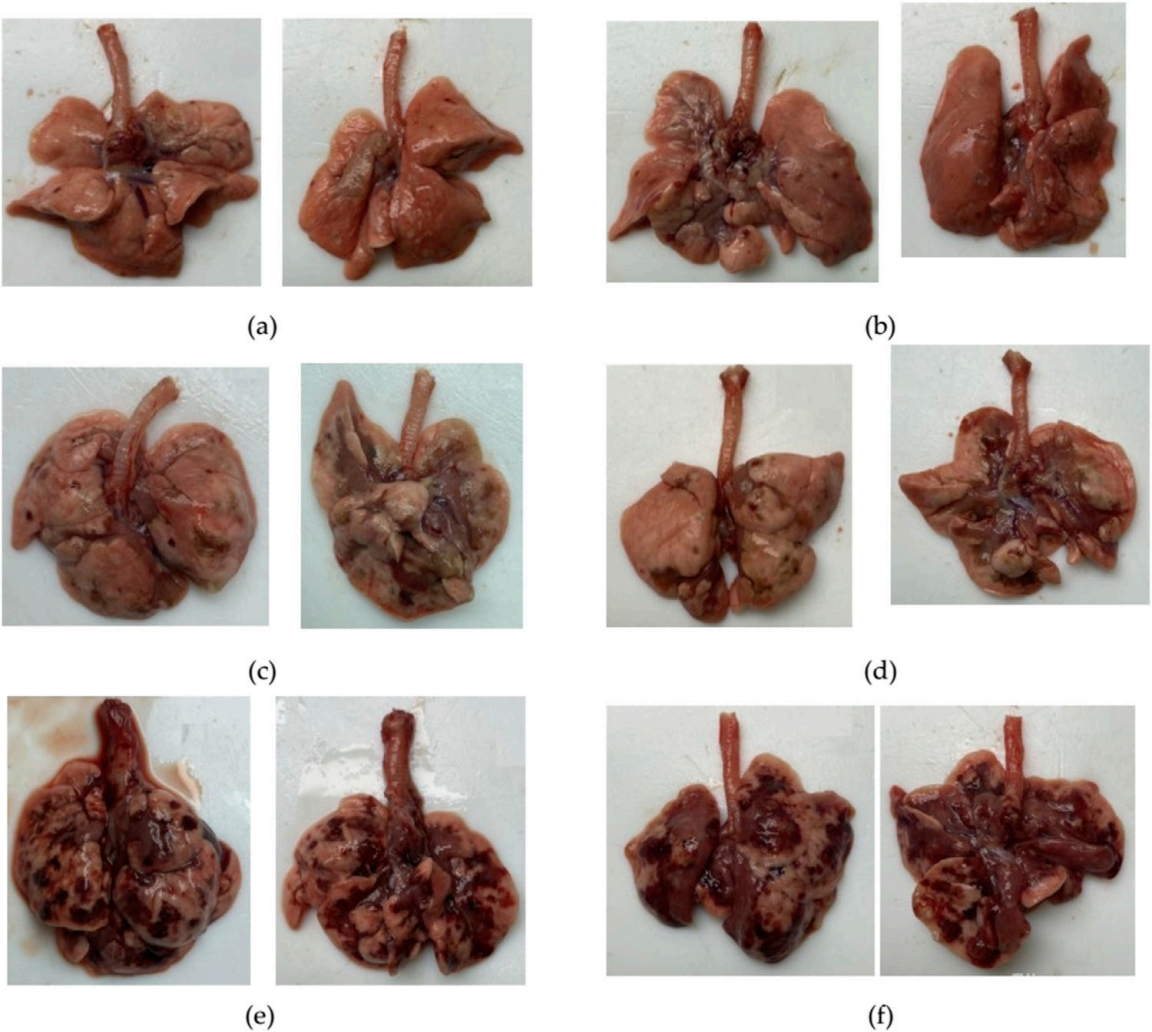
Photographs of the anterior and posterior surfaces of the lungs of animals after modeling PF. **(a)** Lungs on the 22nd day after BLM administration at a dose of 1.5 mg/kg. **(b)** Lungs on the 22nd day after BLM administration at a dose of 2.5 mg/kg. **(c)** Lungs on the 22nd day after BLM administration at a dose of 3 mg/kg. **(d)** Lungs on the 22nd day after BLM administration at a dose of 5 mg/kg. **(e)** Lungs of an animal subjected to unscheduled euthanasia due to cachexia on the 14th day after BLM administration at a dose of 3 mg/kg. **(f)** Lungs of an animal subjected to unscheduled euthanasia due to cachexia on the 14th day after BLM administration at a dose of 5 mg/kg. For animals receiving BLM at a dose of 1.5 mg/kg, visual abnormalities in the lungs could not be recorded in photographs as the changes were visually poorly distinguishable.

Animals receiving BLM had visually evident lung lesions, as shown in Table 5; Figure 7. The severity of these lesions increased with increasing BLM doses and was the lowest for animals receiving 0.5 mg/kg BLM and the highest for animals receiving 5 mg/kg BLM. In addition, two animals in the 3 mg/kg and 5 mg/kg BLM groups that developed cachexia and underwent unplanned euthanasia had significantly more severe lung lesions than all other animals, as visually assessed.

**TABLE 5:** Description of lung lesions detected by visual assessment at necropsy.

Group	Description of changes
Saline	Normal condition of the lungs, no changes
BLM 0.5 mg/kg	Minor, mild changes in one or both lungs in the form of barely noticeable, pinpoint foci of color change to gray, or a visual slight enlargement of one or both lungs
BLM 1.5 mg/kg	Not a very large number of pronounced red punctate lesions and small to medium-sized lesions that change color to gray in both lungs
BLM 2.5 mg/kg	Average quantity of pronounced red punctate lesions and small to medium-sized lesions that change color to gray in both lungs
BLM 3 mg/kg	Expressed quantity of pronounced red punctate lesions and small to medium-sized lesions that change color to gray in both lungs
BLM 5 mg/kg	A large number of pronounced red punctate lesions and small to medium-sized lesions that change color to gray in both lungs

### 3.9 Histological assessment of the lungs

**FIGURE 8:**
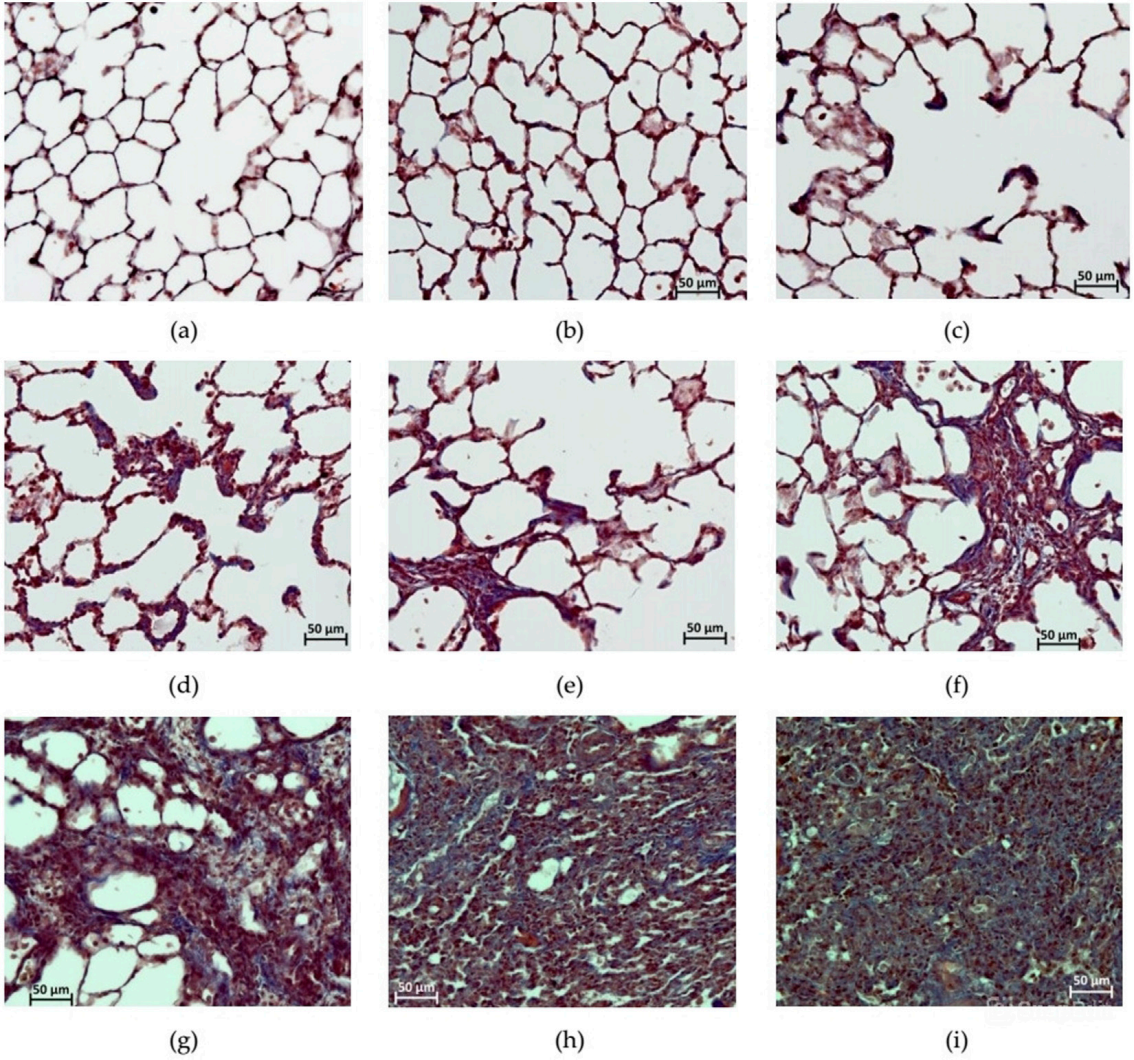
Photomicrographs of histological preparations of the lungs stained with Masson trichrome dye, at 200x magnification, corresponding to the score according to the modified Ashcroft scale. **(a)** Score 0: no deviations from the norm; **(b)** score 1: minor isolated fibrous changes in the inter-alveolar septa (less than three times thicker than normal) and the histoarchitecture of the lungs is not affected; **(c)** score 2: obvious fibrous changes in the inter-alveolar septa (>3 times thicker than normal) with a nodular formation, but not connected to each other, the alveoli are enlarged in size, and the histoarchitecture of the lungs is not affected; **(d)** score 3: adjacent fibrously altered walls of inter-alveolar septa (septum >3 times thicker than normal) mainly throughout the field of view, where alveoli are significantly enlarged in size and histoarchitecture of the lungs is not altered; **(e)** score 4: single fibrous formations (≤10% of the microscope field of view); **(f)** score 5: confluent fibrous changes occupying more than 10% but less than 50% of the microscope field of view, and the lung structure is significantly altered but still preserved; **(g)** score 6: large adjacent fields of fibrous tissue occupying more than 50% of the microscope field of view, and the histoarchitecture of the organ is mostly not preserved; **(h)** score 7: alveoli are erased by fibrous masses, but single air fragments still remain; **(i)** score 8: in the field of view of the microscope, there is complete obliteration of the lung parenchyma by fibrous masses.

Figure 8 shows micrographs reflecting the gradation of the severity of PF, which was assessed in preparations stained with Masson’s trichrome dye, according to the modified Ashcroft scale (Hübner et al., 2008). From a histological analysis perspective, it was appropriate to consider the data from individual animals in groups 5 and 6 that underwent unscheduled euthanasia on the 14th day due to cachexia together with the data from other animals that underwent planned euthanasia on the 22nd day after BLM administration.

**FIGURE 9:**
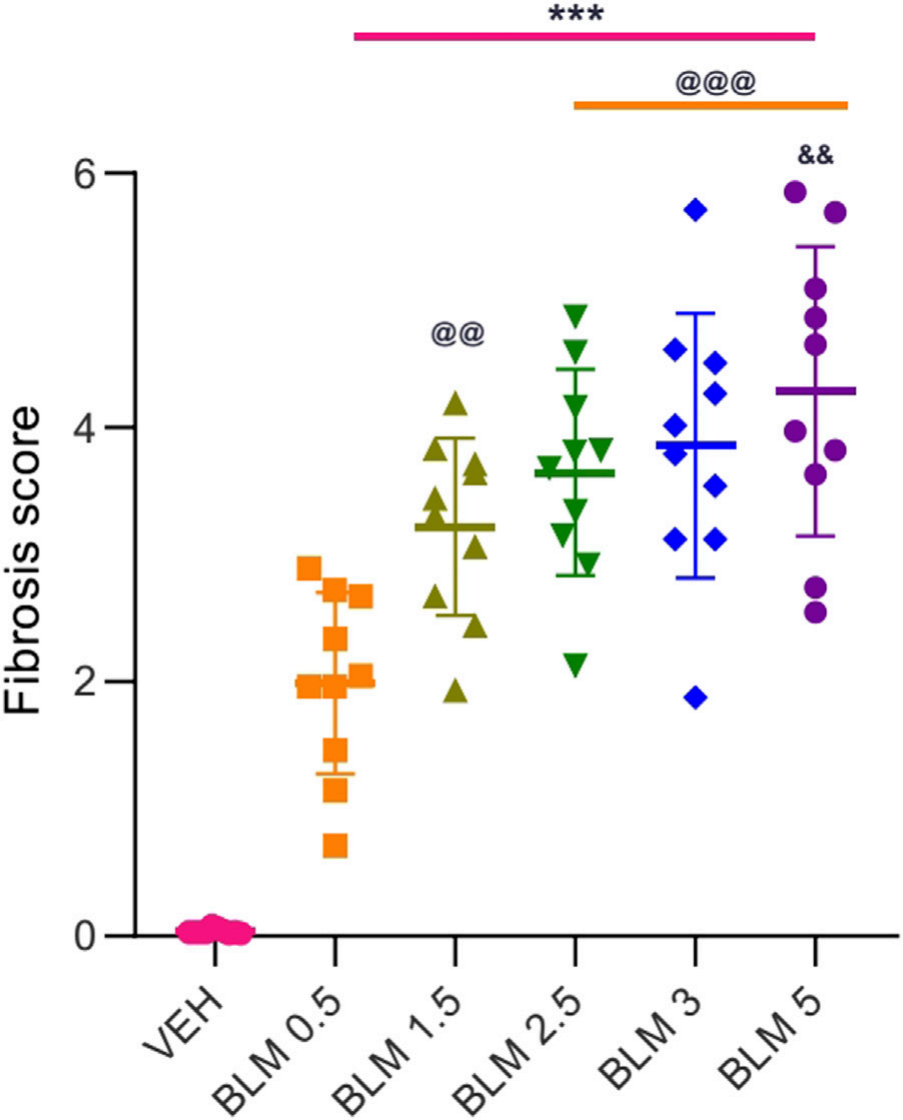
Score semi-quantitative assessment according to the 8-point Ashcroft scale of the severity of fibrosis in the lungs of SD males on the 22nd day after a single intratracheal administration of BLM at different doses. *** ≤0.001 relative to saline; @@ ≤ 0.01 and @@@ ≤ 0.001 relative to BLM 0.5 group at a dose of 0.5 mg/kg; ^&^ р≤ 0.05 relative to BLM 1.5 mg/kg according to one-way ANOVA with *post hoc* Duncan’s test. Data are presented as the MEAN ± SD. For all groups, N = 10.

**FIGURE 10:**
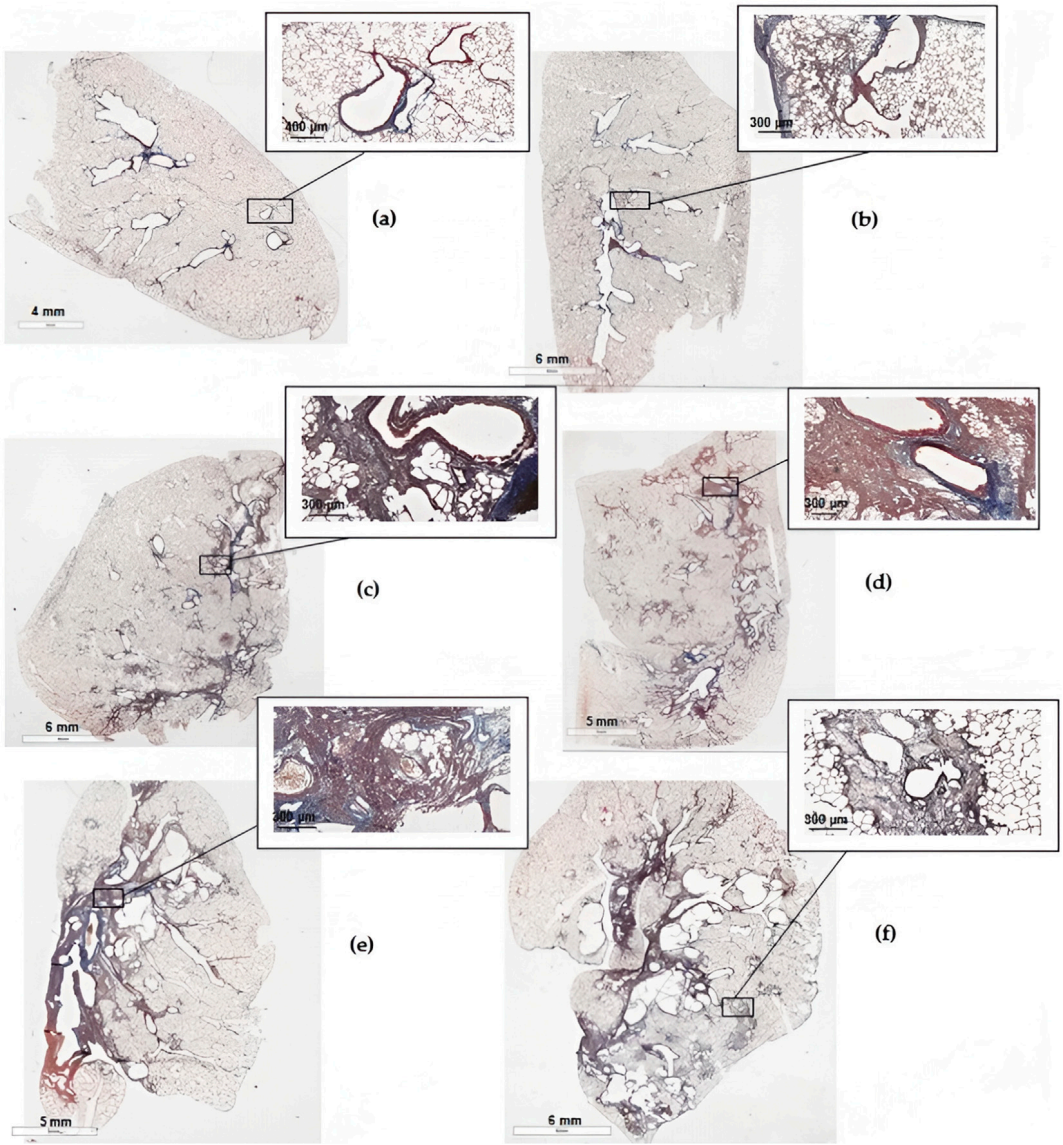
Scans of histological sections of the left lung of animals on the 22nd day after the introduction of BLM in different doses. **(a)** Control saline; **(b)** BLM 0.5 mg/kg; **(c)** BLM 1.5 mg/kg; **(d)** BLM 2.5 mg/kg; **(e)** BLM 3 mg/kg; **(f)** BLM 5 mg/kg (KF-PRO-020-HI, China, magnification is indicated on the scale bar).

After a single intratracheal administration of BLM with subsequent hyperventilation of the lungs, focal PF phenomena were observed in all animals; the foci of fibrous tissue were most often localized in the hilar peribronchial spaces, less often in the pulmonary acini, and only in isolated cases subpleurally. The severity of PF had a clear dose-dependent character (Table 6; Figures 8–10).

**TABLE 6:** Summary of the assessment of changes of degree in the lungs of SD males on the 22nd day after intratracheal administration of BLM at different doses.

Group, BLM dose	Modified Ashcroft scale, 0–8 score		
	Minimum value	Average value	Maximum value
Saline, n = 10	0.02	0.04 ± 0.02	0.08
BLM 0.5 mg/kg, n = 10	0.71	2.03 ± 0.71 ***	2.89
BLM 1.5 mg/kg, n = 10	1.93	3.22 ± 0.70 ***@@	4.19
BLM 2.5 mg/kg, n = 10	2.13	3.65 ± 0.81 ***@@@	4.87
BLM 3 mg/kg, n = 10	1.88	3.86 ± 1.04 ***@@@	5.71
BLM 5 mg/kg, n = 10	2.55	4.29 ± 1.14 ***@@@&	5.85

*** ≤0.001 relative to saline; @@ ≤ 0.01 and @@@ ≤ 0.001 relative to BLM 0.5 group; & ≤ 0.05 relative to the BLM 1.5 at a dose of 1.5 mg/kg according to one-way ANOVA with post hoc Duncan’s test. Data are presented as the MEAN ± SD.

For animals in which the left lung was used for histological evaluation in addition to the right lung, left lung score data were calculated along with the right lung scores, as the average score for them corresponded to the average score for the group calculated for the right lungs.

## 4 Discussion

In our study, after the intratracheal administration of BLM in all doses, PF developed, but the severity of its manifestations was dose-dependent, worsening with an increase in the BLM dose. In humans, body weight is an important parameter characterizing the severity of lung damage in interstitial pneumonia and chronic obstructive pulmonary disease (COPD), leading to the development of PF. Severe lung damage in humans leads to the development of pulmonary cachexia (Schols, 2002), which is characterized by a significant, progressive decrease in body weight and is a risk factor for mortality in patients with COPD (von Haehling and Anker, 2014). In our study, a decrease in body weight was observed in all animals after modeling PF, and the higher the BLM dose, the longer and more pronounced this decrease was. It has been shown that in patients suffering from IPF with a decrease in body weight, the risk of mortality increases (Comes et al., 2022). We also observed the same in our study, when after the introduction of BLM at doses of 3 and 5 mg/kg, cachexia incompatible with life developed in the animals. In healthy SD rats aged 2–3 months, a steady increase in body weight was observed, which we also observed in the control animals (Brower et al., 2015). In animals with PF in our study, the body weight gain rate significantly decreased. A similar decrease in body weight gain has been observed in other studies on animals with PF (Li et al., 2024; Xie et al., 2023; Chu et al., 2019; Gul et al., 2023). However, body weight is not always recorded in studies on animals with PF (Kadam and Schnitzer, 2024a; Zeng et al., 2023; Qu et al., 2020), which we consider a major omission, as body weight and body weight gain are the most important parameters illustrating the degree of progression of lung damage and predicting the outcome of the disease (Pugashetti and Oldham, 2022; Pugashetti et al., 2018; Kulkarni et al., 2019); for example, a two- to three-fold increase in 2-year mortality after the diagnosis of IPF in patients with weight loss has been observed (Nakatsuka et al., 2018). In our study, we showed that the decrease in body weight and the rate of body weight gain were dose-dependent, worsening with an increase in the dose of the damaging agent BLM, which can be used by research workers to make the most informed choice of the BLM dose depending on the objectives of the study. External respiratory function in PF worsens in patients with lung damage, particularly in case of PF (Kulkarni et al., 2019). Increased respiratory rate and decreased vital capacity of the lungs are observed in patients with PF (Flaherty et al., 2000; Weissman et al., 2003; Doherty et al., 1997), as in our study. The dynamics of changes in the respiratory parameters has a pronounced predictive power in relation to the outcome of the disease (Ley et al., 2011; Fujita et al., 2022). In our study, an increase in the respiratory rate and a decrease in the tidal volume of the lungs act as prognostic indicators in relation to the severity of lung damage with an increase in the dose of the damaging agent—BLM. The analysis of BALF in our study showed dose-dependent neutrophil infiltration, which indicates a pronounced inflammatory process. The effect of inflammation on the process of fibrosis development is actively studied. Currently, the leading paradigm for the development of fibrosis is considered to be the imbalance between the destruction of the alveolar endothelial matrix and the healing of this matrix, that is, persistent lung damage and ineffective epithelial healing. Thus, the inflammatory process persists and resolves through apoptosis and phagocytosis of inflammatory and mesenchymal cells. Normally, during recovery from injury, the normal pulmonary architecture is restored. However, in PF, persistent inflammation and impaired recovery lead to intensive development of fibrous tissue and disruption of the pulmonary architecture (Coward et al., 2010). Neutrophil infiltration has been shown to influence the development of PF through enzymes that promote matrix degradation, such as neutrophil elastase (NE), the level of which is increased in the lung parenchyma of patients with PF (Obayashi et al., 1997). NE is a destructive elastase that attacks the extracellular matrix and modulates inflammation and tissue remodeling (Pol et al., 2017). In animals with targeted deletion of the ne−/− gene or after specific inhibition of NE, the development of fibrosis is reduced in a mouse model of PF (Coward et al., 2010; Takemasa et al., 2012; Chua et al., 2007; Taooka et al., 1997; Gregory et al., 2015). In our study, the neutrophil content in the BALF increases with the BLM dose and is associated with worsening of PF, which is consistent with what was described above regarding neutrophil infiltration and the effect of NE on the severity of PF. The increase in lung mass in our study is a characteristic of PF and occurs due to diffuse or multifocal lesions associated with infiltration by inflammatory cells, destruction of the alveolar endothelial matrix, and thickening and fibrosis of the alveolar walls associated with increased collagen deposition (Ge et al., 2024; Cowley et al., 2019). A typical picture of fibrotic lung damage is the presence of fibrotic foci—clusters of active fibroblasts and myofibroblasts—hyperplastic alveolar epithelial cells type 2, whose interconnections with mesenchymal cells enhance the pro-fibrotic environment through the increased activity of growth factors such as TGF-β1, PDGF, and Wnt. Cellular interactions in fibrotic foci lead to excessive production of the intercellular matrix and an imbalance between the deposition and degradation of collagen in favor of the former (Sgalla et al., 2018). The severity of PF is related to the collagen content in fibrotic foci. To quantitatively determine collagen in lung tissue in laboratory animals, the content of hydroxyproline is measured—a degradation product of type-1 collagen, which is the main component of fibrous tissue that forms the basis of fibrous foci in PF (Von Haehling and Anker, 2014; Xu et al., 2019; Ju et al., 2022). According to the American Thoracic Society/European Respiratory Society/Japanese Respiratory Society/Asociación Latinoamericana de Tórax (ATS/ERS/JRS/ALAT) guideline published in 2022, the severity of PF lesions in humans is visualized using high-resolution computed tomography. There are differential signs that indicate the diagnosis of PF and assess its presence and severity, such as honeycombing and/or traction bronchiectasis (Raghu et al., 2022). The same guideline suggests performing the transbronchial lung cryobiopsy (TBLC) procedure to verify the diagnosis of PF. In this study, we allow to establish the histological picture of fibrosis in lung samples taken from different lobes and locations (Troy et al., 2020). However, as the procedure is performed during life and taking biopsy samples has physiological limitations, the level of evidence for the results of this diagnostic method is not high (Raghu et al., 2022). In addition, lung biopsy may not be suitable for patients with severe respiratory dysfunction, pulmonary hypertension, and uncorrectable risk of bleeding (Aburto et al., 2020; Hutchinson et al., 2016; Lentz et al., 2017). In any case, the diagnosis of IPF in humans is possible only with a combination of lung tissue damage visualization techniques, namely, HRCT and histological data obtained during lung biopsy (Rajan et al., 2023). In animal models of PF, visualization of lung tissue fibrosis is performed during postmortem lung histology, with the lungs being assessed as a whole, unlike human lungs during in-life biopsy. In histological preparations stained with Masson trichrome dye (a variant of three-color staining of micropreparations that allows differentiation of collagen fibers, fibrin, muscle tissue, and erythrocytes), the degree of PF expression is assessed using the Ashcroft scale (Hung et al., 2022; Kadam and Schnitzer, 2024a; Liang et al., 2024; Ge et al., 2024; Abd-Elmawla et al., 2023; Mansouri et al., 2019). Our study showed a gradient development of PF, expressed in the scoring of histological preparations; with an increase in the BLM dose, the average score on the Ashcroft scale also increases.

## 5 Conclusion

The presented study demonstrates a dose-dependent effect of BLM on the severity of PF. With an increase in the BLM dose, the general condition of the animals worsens dose-dependently, the function of external respiration worsens, neutrophilic infiltration in the BALF increases, and dose-dependent increase in the average histological score characterizing the degree of PF severity occurs. The least pronounced manifestations of PF were observed when using BLM at a dose of 0.5 mg/kg, and the most pronounced manifestations were observed when using BLM at a dose of 5 mg/kg. These results are addressed to research workers planning to use the BLM-induced PF model to orient themselves in the required degree of PF severity and, accordingly, in the required BLM dose. In addition, our results were obtained using widely used and inexpensive methods available to most laboratories working with preclinical studies on rodents.

## Data Availability

The raw data supporting the conclusions of this article will be made available by the authors, without undue reservation.
